# HDAC1 was involved in placental breast cancer resistance protein regulation in vitro: A preliminary study

**DOI:** 10.1111/jcmm.14414

**Published:** 2019-05-22

**Authors:** Hongyu Duan, Kaiyu Zhou, Yi Zhang, Peng Yue, Tao Wang, Yifei Li, Dajian Qiu, Yimin Hua, Chuan Wang

**Affiliations:** ^1^ Department of Pediatric Cardiology West China Second University Hospital, Sichuan University Chengdu China; ^2^ The Cardiac development and early intervention unit, West China Institute of Women and Children’s Health West China Second University Hospital, Sichuan University Chengdu China; ^3^ Key Laboratory of Birth Defects and Related Diseases of Women and Children (Sichuan University), Ministry of Education Chengdu Sichuan China; ^4^ West China Medical School of Sichuan University Chengdu China

## 1 INTRODUCTION

Placental breast cancer resistance protein (BCRP, encoded by *ABCG2* gene), predominantly located on apical brush‐border membrane of the syncytiotrophoblast, plays a critical role in controlling transplacental transfer rates of various drugs.^1^ Investigations on placental BCRP regulation are most likely to offer great promise for progress in individualized and safe pharmacotherapy during pregnancy. Currently, data regarding epigenetic regulation of placental BCRP are lacking. As core members of class Ⅰ histone deacetylases (HDACs), HDAC1, 2 and 3 are abundantly expressed in trophoblast cells and involved in an extremely broad spectrum of gene regulation in the placenta.^2^, ^3^, ^4^ Recent work in tumour cells,^5^, ^6^, ^7^ along with our previous findings in the placenta,^8^ implied that HDAC1/2/3 might play a significant role in placental BCRP regulation. Importantly, given the discovery of dietary bioactive compounds that can affect HDAC1/2/3 expression and activity,^9^ it will be possible to clinically manipulate placental BCRP safely and effectively by means of epigenetics during pregnancy. Therefore, we carried out this preliminary study to investigate the effect of HDAC inhibition on placental BCRP expression and to further determine the role of HDAC1/2/3 in the placental BCRP regulation in vitro, which might shed some light on the pathway of placental BCRP regulation from the perspective of epigenetics

## 2 MATERIALS AND METHODS

After being authenticated by specific biomarkers (CK7, hPL and hCG positive; HLA‐G negative) to confirm its cell identity,^10^ the human trophoblast BeWo cells were treated with a validated HDAC inhibitor—trichostatin (TSA) at different concentration gradients of 0.5, 1.0, 3.0 and 5.0 μmol/L or transfected with HDAC1/2/3 specific siRNA respectively. After 24–48 hours of exposure, cells were harvested for real‐time quantitative PCR (qRT‐PCR), Western blot, immunofluorescence and efflux activity detection respectively. Data were presented as means ± standard error of mean (SEM) analysed using SPSS 17.0 version (SPSS, Chicago, IL). The significance of difference between the two groups was assessed using the independent sample *t* test. Multiply comparisons were made with analysis of variance (ANOVA) followed by Tukey's honestly significant difference multiple range tests. A 2‐tailed *P* ＜ 0.05 was considered statistically significant. The methods are shown in the ‘Supplemental Materials’ in detail.

## 3 RESULTS

**Figure 1:**
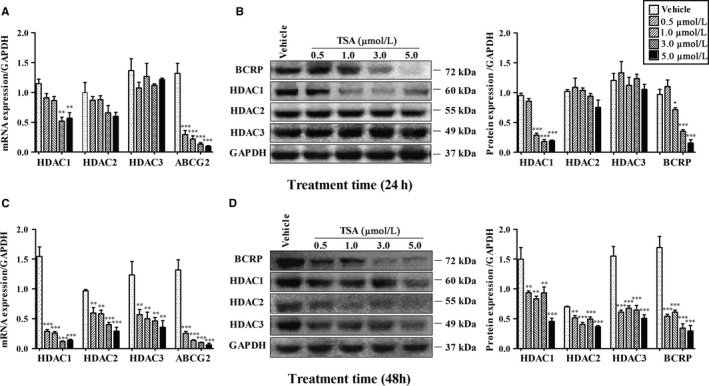
Trichostatin regulation of HDAC1/HDAC2/HDAC3/BCRP mRNA and protein expressions after 24 (A and B) and 48 hours (C and D) of incubation in BeWo. Differences among different groups were assessed using one‐way ANOVA followed by Tukey's honestly significant difference multiple range test. N = 3 for each group. Data are presented as Means ± SEM. *P ＜ 0.05, **P ＜ 0.01, ***P ＜ 0.001

As Figure 1 shows, compared to vehicle groups, after 24 hours treatment of TSA, *HDAC1* mRNA levels in BeWo cells were significantly decreased at concentrations 3.0 μmol/L and 5.0 μmol/L accompanied with prominent reduction of *ABCG2* mRNA expression at every concentration exposure (*P* ＜ 0.01), whereas *HDAC2/3* mRNA was not altered (*P* ＞ 0.05) (Figure 1A). Following 48 hours of exposure, TSA exposure caused inhibition of *HDAC1/2/3* and *ABCG2* mRNA at all concentration gradients (*P* ＜ 0.01) (Figure 1C). A similar effect of TSA on HDAC and BCRP protein expressions was observed (Figure 1B/D). TSA reduced both HDAC1 and BCRP protein levels at concentrations of 1.0/3.0/5.0 μmol/L or that of every concentration after 24 hours (Figure 1B) or 48 hours (Figure 1D) of exposure respectively (*P* ＜ 0.05). As for HDAC2 and HDAC3, such treatment resulted in no significant alterations at 24 hours (Figure 1B) (*P* ＞ 0.05), but dramatic repression was observed at 48 hours (Figure 1D) (*P* ＜ 0.01).

**Figure 2:**
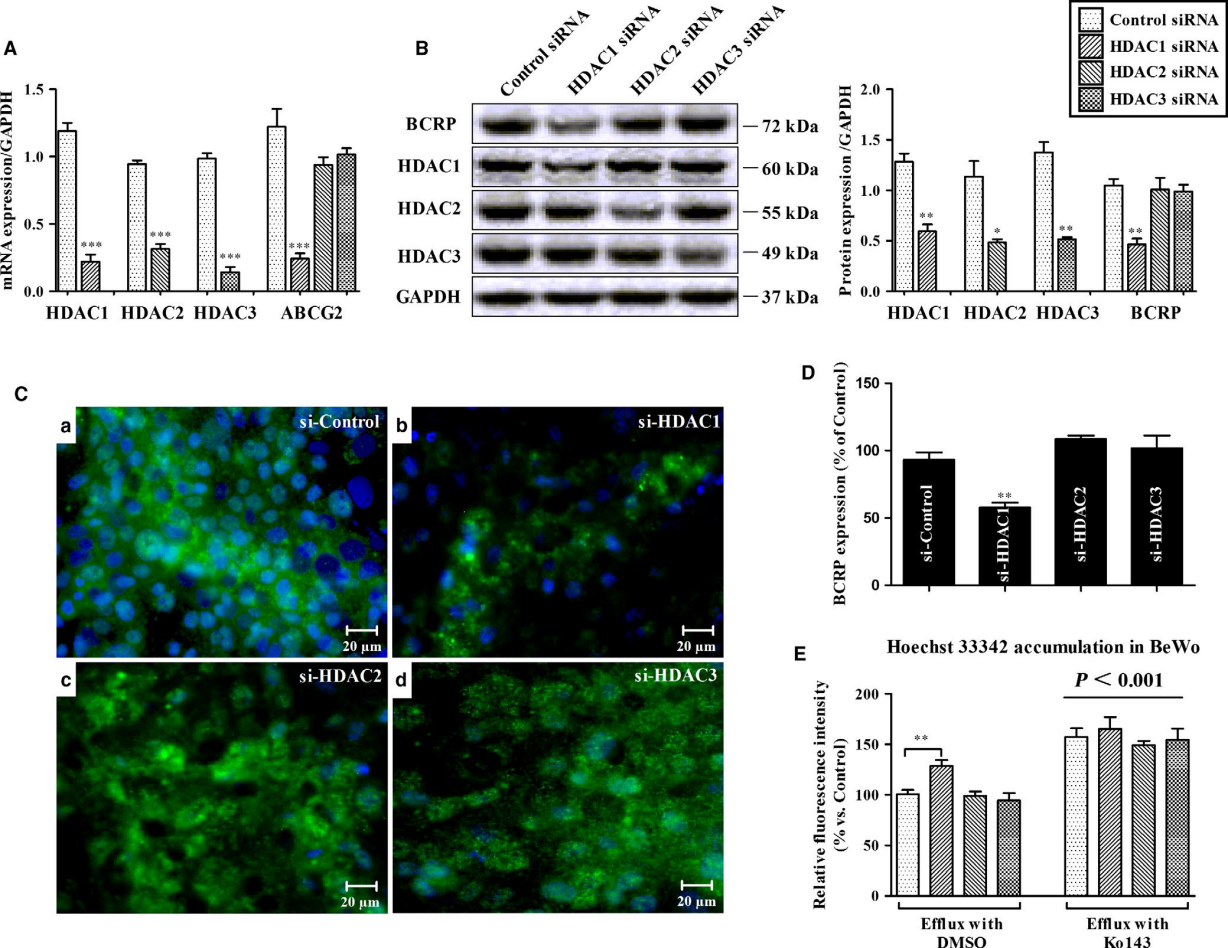
Effect of HDAC1/HDAC2/HDAC3 silencing on breast cancer resistance protein (BCRP) expression and functional activity in BeWo cells. After 48 hours of transfection, the mRNA (A) and protein levels (B) were analysed using qRT‐PCR and Western blot respectively. Representative fluorescent images of BCRP (green staining) were obtained using immunofluorescence microscopy (C). Quantitative fold changes in BCRP expression were analysed using Image J version 1.44 (D). BCRP functionality was measured by the intracellular retention of Hoechst 33342 (5 μg/mL) in the presence or absence of the BCRP‐specific inhibitor Ko143 (1 μmol/L). Intracellular fluorescence was analysed by the fluorescence plate reader (E). Data are expressed as Means ± SEM. *P ＜ 0.05, **P ＜ 0.01, ***P ＜ 0.001; asterisk represents differences among siRNA‐transfected groups; line represents differences between efflux in absence of Ko143 and in presence of Ko143

As Figure 2 shown, after 48 hours of transfection of HDAC1/2/3 siRNA into BeWo cells, endogenous expressions of HDAC1/2/3 were successfully repressed respectively (Figure 2A/B) compared to control groups (all *P* ＜ 0.05). The decline of HDAC1 generated a noticeable decrease in BCRP mRNA and protein production, compared with the control (*P* ＜ 0.01). However, no significant differences in BCRP expression were noted after HDAC2/3 silencing (*P* ＞ 0.05). A consistent decrease in fluorescence of BCRP (in green) was detected in HDAC1 siRNA‐transfected cells (*P* ＜ 0.01) (Figure 2C/D). Figure 2E illustrated the intracellular accumulation of Hoechst 33342 in BeWo after transfections in the absence (namely ‘Efflux with DMSO’) or presence of Ko143 (namely ‘Efflux with Ko143’). Two‐way ANOVA analysis showed that Ko143 exposure (used as a positive control) increased Hoechst 33342 retention at all groups in comparison with the efflux in the absence of Ko143 (*P* ＜ 0.001). Notably, in line with the repression of BCRP expression, transfection of HDAC1 siRNA elevated accumulation of Hoechst 33342 as compared to the control (*P* ＜ 0.01).

## 4 DISCUSSION

To our knowledge, our study was the first one to elucidate that HDAC1 was most likely to be involved in the positive regulation of placental BCRP expression and functionality in vitro. This finding contrasted with the best‐documented and most common biological function of HDAC1, acting as a transcriptional repressor by histone modifications to regulate downstream genes.^5^ However, it was also previously documented that HDAC1 could modify non‐histone proteins and function as a positive regulator of target gene expression either directly or as a coactivator in multiprotein complexes.^11^, ^12^ On account of cell‐specific regulation and individual roles in respective gene regulation of HDACs, the regulatory pathway of HDAC1 on placental BCRP still needs to be further clearly elucidated, which could provide more novel therapeutic targets for controlling drug delivery across the placenta.

Notably, although HDAC1 was found to be involved in BCRP regulation through specific siRNA methodology, some divergent results were found using the methodology of TSA inhibition. For instance, down‐regulation of *ABCG2* mRNA occurred, whereas none of the altered *HDAC1/2/3* mRNA was observed after 0.5 and 1.0 μmol/L TSA exposure at 24 hours (Figure 1A), implying that TSA seemed to inhibit BCRP expression not only through HDAC1‐mediated pathway but also likely through certain undefined‐regulator dependent manners (eg, other HDAC isoforms or non‐HDAC regulation factors), owing to its broader influence on regulatory genes in comparison with siRNA methodology. Additionally, the inconsistent tendency of BCRP mRNA and protein (Figure 1A/B) seems to be somewhat dependent on half‐life, stability or abundance of the protein,^13^ which may confer the delayed reduction of BCRP protein level.

Some limitations of the present study must be considered. Firstly, as non‐syncitialized BeWo cells primarily consist of cytotrophoblast that most appropriately model early gestation, extrapolation of these data from this preliminary study to the whole gestational stage is difficult and more experimental models (ie, syncytializing and/or syncytialized trophoblast cells, primary placental cells, animal models, etc) will definitely be needed to validate our results. Additionally, apart from suppression of placental BCRP, HDAC1 inhibition could result in potential effect on other placental and non‐placental targets,^14^, ^15^ which might have major phenotypic implications. Therefore, more studies should be further undertaken to verify the safety of HDAC1 inhibition, particularly for foetal development. Moreover, given some inconsistent or divergent results between TSA and siRNA methodologies, future studies are therefore required to clarify the effect of TSA on placental BCRP expression or function, aiming to further testify the current data and more importantly, to uncover some regulators besides HDAC1/2/3 involved in this process.

Despite these limitations, we took the first step and made some preliminary exploration of placental BCRP regulation from the perspective of epigenetics in vitro, which was currently lacking in the literature. Obviously, these data obtained from current pilot experiments cannot fully reflect the physiological and biochemical changes in placenta; However, our present findings might expand the limited knowledge with respect to epigenetic regulation of placental BCRP and provide some clues for further studies.

## CONFLICT OF INTEREST

None of authors have declared any conflict of interests.

## AUTHORS CONTRIBUTIONS

Hongyu Duan and Chuan Wang conceived and designed the experiments. Hongyu Duan, Peng Yue, Tao Wang and Yifei Li performed the experiments and analysed the data. Hongyu Duan, Dajian Qiu and Yi Zhang drafted the manuscript. Chuan Wang, Kaiyu Zhou and Yimin Hua revised the manuscript.

## DATA AVAILABILITY STATEMENT

I confirm that my article contains a Data Availability Statement even if no data is available (list of sample statements) unless my article type does not require one (e.g., Editorials, Corrections, Book Reviews, etc.). I confirm that I have included a citation for available data in my references section, unless my article type is exempt.
