# Supplementary material for: HDAC1 was involved in placental breast cancer resistance protein regulation in vitro: A preliminary study
Source: J Cell Mol Med. 2019 May 22;23(8):5818–21. doi: 10.1111/jcmm.14414 (PMC6653152; doi:10.1111/jcmm.14414)
Supplement: Supplementary file 1 [file JCMM-23-5818-s001.doc]

**Materials and methods**

**Cell line, culture conditions**

BeWo cells (human placenta choriocarcinoma) purchased from the Cell Bank of Chinese Academy of Science were cultured in DMEM/F-12 medium (Thermo Fisher Scientific) supplemented with 10% fetal bovine serum, 100 units/mL penicillin and 100 μg/mL streptomycin (Gibco) in a humidified atmosphere of 95% air and 5% CO_2_ at 37 ℃ for 2~4 days.

**Drug treatment**

Cells were grown in six-well plates until 70~80% confluence was reached. Then, the medium was replaced with 2 mL fresh DMEM/F-12. A validated HDAC inhibitor—trichostatin A (TSA) (WXBC0707V, Vetec) was added per well in a volume of 1 μL, 2 μL, 6 μL, and 10 μL, corresponding to the desired concentrations of 0.5, 1.0, 3.0, and 5.0 μM, respectively. Next, 9 μL, 8 μL, 4 μL, and 0 μL of DMSO, applied as a TSA solvent, were individually administered to respective well to ensure an equivalent concentration of DMSO (0.5%) in different groups whilst culture medium with DMSO (0.5%) was used as vehicle. The medium was replaced daily with freshly prepared TSA- or vehicle-containing medium, and the cellular status was evaluated as well. Considering that TSA treatment for 72 h induced a marked decrease of cell viability in BeWo cells as we previously described,^1^ the cells were harvested at 24/48h for next step tests after being washed with ice-cold PBS. All experiments were repeated three times.

**Transfection of HDAC 1/2/3 small interfering RNA**

After cells were grown to 70~80% confluence, 80 nM small interfering RNA (siRNA) specific for HDAC1 (stB0001570A, GuangZhou RiboBio. Co., China)/HDAC2 (stB0001571A, GuangZhou RiboBio. Co. China)/HDAC3 (stB0001590A, GuangZhou RiboBio. Co. China) or control siRNA (siN05815122147, GuangZhou RiboBio. Co., China) was transfected into BeWo cells using Lipofectamine^R^ RNAiMAX Reagent (13778-150, Invitrogen) according to the manufacturer’s instructions. The siRNA concentration for transfection was selected from the previously described protocol to achieve a satisfactory transfection efficiency.^2^ After 48 h of transfection, cells were harvested for next step tests. All experiments were repeated three times.

The siRNA sequences used were as follows:

siRNA HDAC1 Sense: 5'-GCGACUGUUUGAGAACCUU dTdT-3'
 Anti-sense: 3'-dTdT CGCUGACAAACUCUUGGAA-5'

siRNA HDAC2 Sense: 5'-CCGUAAUGUUGCUCGAUGU dTdT-3'

Anti-sense: 3'-dTdT GGCAUUACAACGAGCUACA-5'

siRNA HDAC3 Sense: 5'-GAGCAACCCAGCUGAACAA dTdT-3'

Anti-sense: 3'-dTdT CUCGUUGGGUCGACUUGUU-5'

**Real-time quantitative PCR analysis**

Total RNA was isolated and purified using the Trizol reagent (Invitrogen, Life technologies, Carlsbad, CA). RNA concentration and purity were assessed by a A260/A280 ratio spectrophotometrically using the Nanodrop_2000 instrument (Thermo Scientific). RNA integrity was determined by agarose gel electrophoresis using the 28S/18S rRNA ratio. RNA (1 μg) was reverse transcribed into cDNA using PrimeScript^TM^ RT Reagent Kit with gDNA eraser (RR0047A, Takara, Japan) according to the manufacturer's instructions.

Amplification of cDNA was performed with SsoFast EvaGreen Supermixture (Bio-Rad Laboratories, Hercules, CA) using 5 μL reaction mixture, 3 μL nuclease-free H_2_O, 0.5 μL forward primer, 0.5 μL reverse primer and 1 μL cDNA in a final reaction volume of 10 μL. The cycling conditions were as follows: initial denaturation at 95 ℃ for 3 min, followed by 39 cycles of 30 s at 95 ℃, 10 s at 55 ℃ for *HDAC1/HDAC2/HDAC3/ABCG2/GAPDH*, and a continuous melt curve from 65~95 ℃. A validation experiment had been performed in which equivalent amounts of cDNA were used. Different reference genes were assessed for expression stability (M) using normalization strategies under experimental treatment.^3,4^ The reference genes tested were: glyceraldehyde 3-phosphate dehydrogenase (*GAPDH*), beta-2-microglobulin (*B2M*), zeta polypeptide (*YWHAZ*), β-actin, hypoxanthine- guanine phosphoribosyltransferase (*HPRT*). Notably, *GAPDH* was the most stable reference gene guaranting its use as a appropriate internal control for normalization. Moreover, *GAPDH* exhibited similar amplification efficiencies and CT values between the control group and the treatment group. Additionally, we have ascertained the efficiencies of amplifications for all genes (*HDAC1/HDAC2/HDAC3/ABCG2/GAPDH*) in our study, which were consistent across a range of template concentrations. All the slope of the amplification efficiency curves were more than 95% and efficiencies for the target genes (*HDAC1/HDAC2/HDAC3/ ABCG2*) and the internal control (*GAPDH*) were approximately equal (0.957-0.974). All samples were amplified in triplicates. Gene expression was represented for the cycle threshold value (CT) by the mean of triple tests. Data were normalized to expression of *GAPDH* and calculated through 2^-△△Ct^ method.

The primer sequences specific for target genes were as follows:

HDAC1: 5'- CACCCATTCTTCCCGTTCT-3' (forward)

HDAC1: 5'-GCACTTGGCATTTCAGGAGT-3' (reverse)

HDAC2: 5'-GTTCTGGCATCCTCCCTGT-3' (forward)

HDAC2: 5'-TTCCATCTCCTCCATCCACT-3' (reverse)

HDAC3: 5'-GAGGGATGAACGGGTAGACA-3' (forward)

HDAC3: 5'-CAGGTGTTAGGGAGCCAGAG-3' (reverse)

ABCG2: 5'-TATAGCTCAGATCATTGTCACAGTC-3' (forward)

ABCG2: 5'-GTTGGTCGTCAGGAAGAAGAG-3' (reverse)

GAPDH: 5'-GAAGGTGAAGGTCGGAGTC-3' (forward)

GAPDH: 5'-GAAGATGGTGATGGGATTTC-3' (reverse)

**Western blot analysis**

Cells were lysed in RIPA (P0013B, Beyotime, China) containing complete protease inhibitor cocktail (P8340, Sigma-Aldrich) for 20 min at 4 ℃ and centrifuged at 12,000 g for 5 min at 4 ℃. Supernants were analyzed for protein concentration by enhanced BCA protein assay kit (P0010S, Beyotime, China) following manufacturer’s protocol. Cell lysates were boiled in 4×sample buffer for 5 min and 50 μg protein/lane was subjected to 8% SDS-polyacrylamide gel, followed by blotting onto polyvinylidene difluoride membranes (Millipore, Bedford, MA). After blocking for 60 min with 5% nonfat milk in Tris base buffer containing 0.1% Tween 20 (TBST), membranes were incubated overnight at 4 ℃ with primary antibodies against HDAC1 (dilution 1:1000; 10197-1-AP, Proteintech)/HDAC2 (dilution 1:1000; 12922-3-AP, Proteintech)/HDAC3 (dilution 1:500; 10255-1-AP, Proteintech)/BCRP (dilution 1:500; ab130244, Abcam) and GAPDH (dilution 1:500; CW0100A, CWBIO). Following three times of washing with TBST, the membranes were reacted with horseradish peroxidase-conjugated goat anti-mouse/goat anti-rabbit immunoglobulin G (IgG) secondary antibodies (dilution 1:2500) for 2 h at room temperature. Washed thrice in TBST, the immunoreactive bands were visualized by enhanced chemiluminescene detection system. The protein band intensity was quantified by software Gelpro32 and normalized against the GAPDH as an internal control.

**Immunofluorescence staining of placental BCRP**

To further confirm the effect of transfection of HDACs siRNA on placental BCRP, its protein expression was visualized using an immunofluorescence microscopy (Nikon. Eclipse. 80i). Briefly, following 48 h transfection as described above, cells were washed thrice with ice-cold PBS and fixed in 4% formaldehyde. Non-specific binding sites were blocked with 3% of BSA and 2% of fetal bovine serum in 0.2% Triton X-100/PBS. The cells were then washed and incubated overnight at 4 ℃ with primary antibodies against BCRP (ab130244, Abcam) diluted 1:200 in blocking buffer. After the cells were washed with PBS, Fluor 488 goat anti-mouse secondary antibody (A11001, Invitrogen) was added (dilution 1:500) and incubated in the dark for 40 min at room temperature. Cell nuclei were stained with 4’, 6-diamidino-2-phenylindole (DAPI) (Sigma) at 1:500 dilution for 5 min. The slides were washed twice with PBS and fluorescence images were visualized using an immunofluorescence microscopy. Negative controls processed without the primary antibody showed negligible background fluorescence.

Images were processed identically using Adobe Photoshop CS6, and BCRP immunofluorescence for each sample was quantified by Image J version 1.44 software (National Institutes of Health, Bethesda, USA). In brief, the images imported into Image J software were auto-thresholded to binary photos utilizing the “Make Binary” function after being converted to 8-bit images. By employing the “Add to Manager” function, regions of interests (ROIs) were subsequently identified to enclose the cellular localization of BCRP. The “Analyze Particle” function was thereafter used to analyze the areas of ROIs in each of the binary images, and the sum of integral optical density (IOD SUM) of BCRP in cells was calculated after background fluorescence was removed by “Subtract Background” function. Finally, the mean IOD was defined as IOD SUM divided by area.

**Evaluation of placental BCRP functional activity**

The functional efflux activity of placental BCRP was identified by evaluating the intracellular accumulation of a specific BCRP fluorescent substrate—Hoechst 33342 (C1022, Beyotime), in the presence or absence of BCRP specific inhibitor—Ko143 (CS-0298, Chemscene) according to the manufacturer’s instructions. The efflux activity of BCRP was highly sensitive to temperature, functioning optimally near 37 ℃, but effectively inactive at 4 ℃. Briefly, after 48 h of siRNA transfection, BeWo cells were harvested. Cells were suspended at a density of 1.0×10^6^/mL in containers offering protection from light, and were preincubated at 37 ℃ in medium with 5 μg/mL Hoechst 33342 in presence of Ko143 at 1 μM concentration or DMSO (diluent control for Ko143 solution) for 30 min (loading phase). At the end of the loading period, medium was removed and cells were washed twice with dye-free ice-cold PBS (centrifugation for 5 min at 500×g) . Subsequently, cell were resuspended and further incubated at 37 ℃ for an additional 60 min in dye-free warmed medium containing Ko143 or containing DMSO, respectively (efflux phase). Efflux was terminated by pelleting and resuspending the cells in ice-cold PBS. The cells were kept on ice until analysis—low temperatures stopped the efflux reaction and prevented uncontrolled dye efflux. The intracellular fluorescence of Hoechst 33342 was measured by microplate spectrophotometer (Varioskan flash, Thermo scientific) under the excitation wavelength 350 nm and emission wavelength 460 nm.

**Statistical analysis**

All data were presented as means±standard error of mean (SEM) analyzed by SPSS 17.0 version (SPSS, Chicago IL, USA). Shapiro-Wilk test and homogeneity test of variance were used to confirm that quantitative data from different groups were normally distributed and met the homogeneity of variance. The significance of the difference between two groups was assessed by the independent sample *t*-test. Multiply comparisons were made with analysis of variance (ANOVA) followed by Turkey’s honestly significant difference multiple range test. A 2-tailed P value＜0.05 was considered statistically significant.

**References**

1. Duan H, Wang C, Zhou K, et al. The effect of histone deacetylase inhibition on the expression of P-glycoprotein in human placental trophoblast cell lines. *Placenta*. 2017; 49: 37-47.

2. Duan H, Zhou K, Zhang Y, et al. HDAC2 was involved in placental P-glycoprotein regulation both in vitro and vivo. *Placenta*. 2017; 58: 105-114.

3. Bustin SA, Benes V, Garson JA, et al. The MIQE guidelines: minimum information for publication of quantitative real-time PCR experiments. *Clin Chem*. 2009; 55 (4): 611-622.

4. Lye P, Bloise E, Dunk C, et al. Effect of oxygen on multidrug resistance in the first trimester human placenta. *Placenta*. 2013; 34 (9): 817-823.
